# Potentials and limits to enhance cognitive functions in healthy and pathological aging by tDCS

**DOI:** 10.3389/fncel.2015.00355

**Published:** 2015-09-14

**Authors:** Kristin Prehn, Agnes Flöel

**Affiliations:** Department of Neurology and NeuroCure Clinical Research Center, Charité Universitätsmedizin BerlinBerlin, Germany

**Keywords:** transcranial direct current stimulation (tDCS), Alzheimer’s dementia (AD), mild cognitive impairment (MCI), memory, executive control

## Abstract

Transcranial direct current stimulation (tDCS) is a non-invasive brain stimulation technique that is increasingly used in research and clinical settings to enhance the effects of cognitive training. In our present review, we will first summarize studies using tDCS alone and in combination with cognitive training in older adults and patients with Alzheimer’s dementia (AD). We will also review one study (Meinzer et al., 2014c) that showed an improvement in cognitive performance during anodal tDCS over the left inferior frontal cortex in patients with mild cognitive impairment (MCI) which is regarded as a prodromal stage of AD. Although promising short-term results have been reported, evidence from randomized controlled trials (RCTs) with sufficient sample sizes is scarce. In addition, stimulation protocols (in terms of intensity, duration, and repetition of stimulation) that lead to sustained improvements in outcome measures relevant for daily life still remain to be established. Following, we will discuss modulating factors such as technical parameters as well as the question whether there are specific cognitive functions (e.g., learning, memory consolidation, executive control) which are more amenable to tDCS enhancement than others. Finally, we will highlight future directions and limitations in this field and emphasize the need to conduct RCTs to establish efficacy of interventions for activities of daily life for a given patient population.

## tDCS as a Tool for Enhancing Cognition during Healthy and Pathological Aging

Due to the constant growth of the elderly population worldwide, Alzheimer’s dementia (AD) and other neurodegenerative disorders are rising exponentially (Burke and Barnes, 2006; Grady, 2012). Even during normal aging, a significant decrease in cognitive abilities (e.g., learning, memory formation, or executive control) is observed and gradually constrains daily activities and independent living in older adults. Therefore, research efforts need to be devoted to evaluate intervention strategies that counteract or delay the onset of cognitive decline. Given the paucity of pharmacological interventions, strategies for non-pharmacological enhancement are receiving increasing attention, including cognitive training (Belleville, 2008; Jean et al., 2010), physical activity (Kramer and Erickson, 2007), nutritional supplementation (Farias et al., 2014), and non-invasive brain stimulation techniques such as transcranial direct current stimulation (tDCS) and transcranial magnetic stimulation (TMS; Elder and Taylor, 2014; Floel, 2014).

Transcranial direct current stimulation is a non-invasive brain stimulation technique that is increasingly used in research as well as in clinical settings to probe and modulate cortical plasticity. tDCS involves the application of at least two electrodes on the scalp of the participant. One electrode serves as the anode and the other one as the cathode. A continuous week current (0.5–2.0 mA) is applied between the two electrodes and flows from the anode to the cathode. To minimize chemical reactions at the electrode-skin interface, tDCS should be performed with non-metallic, conductive rubber electrodes, covered by saline soaked sponges or rubber electrodes used with conductive gel. As the current flows between the electrodes, it passes through the brain and modulates neural activity underneath the electrodes dependent on the direction, intensity, and duration of the current (Miranda et al., 2006): at currents of 1 mA, tDCS results in depolarization of the neurons underneath the anode, hence causing an excitatory effect, whereas underneath the cathode tDCS causes hyperpolarization and thus inhibition of cortical neurons (Nitsche and Paulus, 2000). Enhancement of tissue excitability under the anode and decreased excitability under the cathode has been found for 1 mA currents and stimulation periods for up to 20 min. A current strength of 2 mA, however, causes excitability increases under both electrodes as demonstrated in a recent study by Batsikadze et al. (2013). The authors specifically showed that tDCS applied with a current strength of 2 mA for up to 20 min over the motor cortex reverses the previously found inhibitory effect under the cathode to facilitation.

Since tDCS always involves the application of an anode and a cathode, terms such as “anodal tDCS” (atDCS) and “cathodal tDCS” (ctDCS) are inaccurate. However, authors use this terminology to indicate that they specifically refer to the effects caused under anode or cathode. When authors use the term “atDCS over the dorsolateral prefrontal cortex (DLPFC),” for instance, they refer in particular to the excitability enhancing effect of tDCS on the DLPFC where the anode has been placed. Likewise, the terminology “ctDCS over the DLPFC” indicates that authors refer in particular to the excitability decreasing effect of tDCS under the cathode. Since in most studies using a cephalic electrode montage one smaller electrode (e.g., 5 cm × 7 cm) is placed over the target brain region, whereas the other electrode is placed over contralateral frontopolar cortex and enlarged (e.g., 10 cm × 10 cm) to render stimulation over this cortex functionally inefficient (Nitsche et al., 2007), the latter electrode is referred to as the “reference” electrode (in contrast to “stimulation” electrode). Some authors also use an extracephalic electrode montage to reduce unintended effects on the brain alltogether. Fertonani et al. (2014), for instance, placed the reference electrode (in this case, the cathode) over the right shoulder while the anode was placed over the left DLPFC.

In contrast to TMS, tDCS does not directly elicit action potentials (e.g., by means of suprathreshold resting membrane potential change) but renders neuronal populations more or less ready to fire in response to additional input. In other words, tDCS changes the likelihood that an incoming action potential will result in post-synaptic firing both immediately during stimulation and a short period of time after stimulation (“after-effects”). Thus, the primary mechanism involved in tDCS is a subthreshold alteration of the resting membrane potential involving ionic concentration shifts within the extracellular fluid, whereas the after-effects of tDCS on cortical excitability involve synaptic plasticity of glutamatergic connections (i.e., *N*-methyl-D-aspartate (NMDA) receptor-dependent processes; Nitsche et al., 2004). With regard to tDCS effects involving synaptic plasticity, animal and human studies have indicated that tDCS also introduces a secondary mechanism (in addition to alterations in resting-membrane potential) that involves the induction of long-term potentiation and depression (LTP and LTD)-like processes (Stagg and Nitsche, 2011). LTP is defined as a mechanism which leads to a long-lasting increase in the efficacy of synaptic communication in specific neuronal pathways (i.e., a strengthening of the pathways) resulting from high-frequency electrical bursts during stimulation. Following LTP induction, a pre-synaptic stimulation induces a “potentiated” post-synaptic response. For example, a pulse of the same intensity now activates a larger set of synapses or the same set of synapses is now activated by a pulse of lower intensity. Since LTP leads to a modulation of synaptic strength that can be maintained for days, months, or even years, this mechanism has been postulated as a candidate for memory formation in the brain. Specifically, it has been proposed that by this mechanism memory traces are laid down in neuronal pathways. If a neuronal pathway is activated to a certain level, a permanent change in that network would occur thereby allowing the information to be more easily retrieved or remembered. For example, practicing a certain behavior such as finger movements during piano playing naturally increases the excitability of neurons in the motor cortex. Increased excitability promotes improvements in task performance by facilitating LTP-like processes between the neurons involved in that task. Therefore, increasing neuronal excitability with brain stimulation, whether direct or indirect, may provide a means of inducing a physiological state supporting the acquisition of novel skills or memory formation (Floel and Cohen, 2010). While LTP occurs underneath the anode, its counterpart LTD, which is defined as an activity-dependent reduction in the efficacy of neuronal synapses, occurs underneath the cathode. Both mechanisms involved in tDCS (alteration of resting membrane potential and LTP/LTD-like synaptic plasticity) are susceptible to pharmacological modulation with dopaminergic and serotonergic agents (Nitsche et al., 2009; Monte-Silva et al., 2010).

With regard to the application of tDCS, two main strategies to alter brain activity (and thereby to improve cognitive functions) can be distinguished. The first strategy aims to increase cortical excitability or training-induced LTP-like mechanisms in a specific brain area of interest (e.g., by placing the anode over that brain region). Since the DLPFC has been found to be involved in working memory (e.g., Owen et al., 2005), increasing cortical excitability in the DLPFC, for instance, may promote performance during a working memory task. As a second strategy, tDCS may also be used to inhibit a certain brain region or brain network (i.e., by placing the cathode over that brain region). This second approach is particularly valuable for neuroscience research to provide causal evidence for the involvement of a specific brain region in a particular task. The inhibition of one brain function might also imply the relative enhancement of another brain function. This principle can also be used to improve brain function in certain settings (e.g., after unihemispheric stroke): in stroke patients, for example, improvements of brain functions can be achieved by enhancing the excitability of the lesioned areas. This can be accomplished by direct excitatory stimulation of the lesioned areas or by inhibitory stimulation of the contralateral and healthy hemisphere which is now after the stroke “over-active.” Placing the cathode in the latter case over the contralateral hemisphere reduces transcallosal inhibition and thus disinhibits the lesioned area (Otal et al., 2015).

A potential limitation of tDCS is its limited spatial accuracy. As the current passes through the brain from anode to cathode and modulates neural activity simultaneously underneath anode and cathode, it can be difficult to relate the effects of tDCS to a specific brain region. It should also be noted that tDCS not only affects the brain regions directly under the electrodes but may also modulate functional connectivity between remote but functionally associated brain areas (Polania et al., 2011) or influence the within network connectivity (Meinzer et al., 2012). Furthermore, it is also important to note that the vast majority of studies investigating the mechanisms of tDCS were conducted by stimulating the primary motor cortex (M1). Thus, it is not clear to what extent these findings are transferable to other areas of the cortex (e.g., those recruited during higher cognitive processes), although it is likely that the mechanisms are similar (Stagg and Nitsche, 2011).

When following established protocols, tDCS is considered safe and does not cause side effects other than discomfort including itching sensations underneath the electrodes or mild headache (Paulus et al., 2008). Since this method is inexpensive and easy to administer, it is also highly suitable for application in patient populations, during training interventions, as well as in home-based settings.

## Review of Recent tDCS Studies on Cognitive Decline in Older Healthy Subjects and Patients with AD and MCI

All aging individuals will develop some degree of decline in cognitive capacities as time progresses. Aging impacts the human brain and all processes that underlie the ability to think and reason (i.e., information processing in general) ranging from a loss of neurons (i.e., a decrease in gray and white matter integrity) and cortical thinning, impaired neurotransmitter-receptor binding and signaling, an accumulation of neurofibrillary tangles and amyloid plaques, or altered concentrations of various brain metabolites (for a review, see Jagust, 2013). Structural changes are accompanied by functional reorganization such as compensatory hyperactivity of neural networks which indicates both reduced neural efficiency and reduced neural specificity (for a review, see Antonenko and Floel, 2014). For instance, it has been shown that task-related prefrontal activity is increased in older adults and patients with cognitive impairment (e.g., Sperling et al., 2009; Elman et al., 2014). This hyperactivity is interpreted as a marker of “compensatory neurocognitive scaffolding” in response to the challenges posed by declining neural structures (i.e., the use of alternative neural circuits; Park and Reuter-Lorenz, 2009). These structural and functional changes in brain architecture may impact mental capacities in various cognitive domains such as attention, processing speed, episodic memory, decision making, executive control functions, emotion processing and regulation.

A number of studies have demonstrated that tDCS can be used to enhance cognitive functions in healthy subjects (for a review, see Shin et al., 2015). Some authors, however, also published negative reports or showed conflicting results (for reviews, see Tremblay et al., 2014; Horvath et al., 2015). Given that one of the major effects of tDCS is the induction of LTP and LTD-like plasticity, which is considered as being the physiological basis of learning and memory processes, tDCS is an attractive method to specifically overcome learning- and memory-related deficits associated with cognitive decline during healthy and pathological aging. Indeed, numerous studies have been conducted in older subjects and patients with AD or mild cognitive impairment (MCI), which will be discussed in the following part of the review. The focus will be put on studies in patients with AD and MCI.

### tDCS Studies in Older Healthy Subjects

In older healthy adults, tDCS has been employed to improve learning (of motor skills; e.g., Lindenberg et al., 2013; Zimerman et al., 2013; Hardwick and Celnik, 2014; Meinzer et al., 2014d), working memory (e.g., Berryhill and Jones, 2012; Park et al., 2014; Jones et al., 2015b), memory formation (encoding, retrieval, and recognition of episodic memories: e.g., Floel et al., 2012; Manenti et al., 2013; Sandrini et al., 2014), declarative memory retrieval (e.g., Holland et al., 2011; Ross et al., 2011; Meinzer et al., 2013; Fertonani et al., 2014), as well as decision making under risk (Boggio et al., 2010) and error awareness (Harty et al., 2014) in a large number of studies. Here, we will discuss in more detail two recent studies from our group aimed at improving object-location memory and semantic word generation, respectively, to give the reader some insight into the specifics of studies in this field and also briefly present several other landmark papers. For an overview of the studies in older healthy subjects, modalities, and stimulation parameters, see **Table 1**, and also a recent meta-analysis by Hsu et al. (2015).

**Table 1:** Overview of tDCS studies in healthy older subjects.

Reference	Number of subjects	Age range in years	Trial type	Modalities	Polarity of stimulation electrode, position	Position of reference electrode^∗^	Current strength, duration, number of consecutive sessions	Outcome
Berryhill and Jones, 2012	*N* = 25	56–80	Randomized cross over within subjects	(Verbal and visual) working memory	atDCS over left or right DLPFC vs. sham	Contralateral cheek	1.5 mA, 10 min	Improved working memory during left and right atDCS (specifically in subjects with higher education)
Boggio et al., 2010	*N* = 28 (9/10 per group)	50–85	Randomized between subjects (three stimulation groups)	Decision making in a gambling task	Bilateral atDCS over prefrontal cortex (left anodal/right cathodal or right anodal/left cathodal) vs. sham	-	2 mA, 15 min	Increased risk taking during right atDCS/left ctDCS
Fertonani et al., 2014	*N* = 40 (20 per group)	61–83	Randomized cross over within subjects (two age groups)	Picture naming (declarative memory retrieval)	atDCS over left DLPFC vs. sham	Right shoulder	2 mA, 5 min during vs. 10 min before task execution	Improved naming performance during atDCS in older subjects, improved performance during and after atDCS in younger subjects
Floel et al., 2012	*N* = 20	50–80	Randomized cross over within subjects	Episodic memory (memory encoding)	atDCS over right temporoparietal cortex vs. sham	Left frontopolar cortex	1 mA, 20 min	Improved episodic memory performance one week after atDCS
Hardwick and Celnik, 2014	*N* = 33 (11 per group: old atDCS, old sham, young sham)	Mean age ± SD in groups with older subjects: 56.3 ± 6.8, 59.6 ± 8.1	Randomized between subjects (three groups)	Procedural memory (finger tapping)	atDCS over ipsilateral cerebellum vs. sham	Ipsilateral buccinator muscle	2 mA, 15 min	Improved motor skill learning in older subjects during atDCS
Harty et al., 2014	*N* = 96 (24 per experiment)	65–86	Randomized cross over within subjects	Error awareness during a Go/No-go task	tDCS (atDCS or ctDCS over left or right DLPFC) vs. sham in four different experiments	Vertex	1 mA, 37.5 min	Improved error detection during atDCS over right DLPFC
Holland et al., 2011	*N* = 10	62–74	Randomized cross over within subjects	Picture naming (declarative memory retrieval)	atDCS over left inferior frontal cortex	Contralateral frontopolar cortex	2 mA, 20 min	Faster naming and decreased neural activity in Broca’s area during atDCS
Jones et al., 2015b	*N* = 72 (18 per group)	55–73	Randomized between subjects (four stimulation groups)	(Verbal and visual) working memory	atDCS over right DLPFC, parietal, or alternating DLPFC/parietal cortex vs. sham	Contralateral cheek	1.5 mA, 10 min, 10 consecutive sessions	Improved working memory in all training groups, maintained improvement one month after atDCS in same and transfer working memory tasks
Lindenberg et al., 2013	*N* = 20	61–77	Randomized cross over within subjects	Cued choice response time task	Unilateral atDCS over left primary motor cortex vs. bilateral atDCS vs. sham	Right frontopolar cortex	1 mA, 30 min	Complex interhemispheric modulations of neural networks during atDCS
Manenti et al., 2013	*N* = 64 (32 per group)	Mean age ± SD: 67.91 ± 4.72	Randomized cross over within subjects (two age groups)	Episodic memory (memory retrieval)	atDCS over left or right DLPFC or parietal cortex vs. sham	Contralateral frontopolar cortex	1.5 mA, 6 min	Improved memory retrieval during atDCS over left DLPFC and parietal cortex
Meinzer et al., 2013	*N* = 20	60–76	Randomized cross over within subjects	Semantic word generation	atDCS over left inferior frontal gyrus vs. sham	Right frontopolar cortex	1 mA, 20 min	Improved semantic word generation (less errors) during atDCS and a more “youth-like” functional network configuration
Meinzer et al., 2014d	*N* = 18	61–77	Randomized cross over within subjects	Semantic word generation, cued choice response time task	Unilateral atDCS over left primary motor cortex vs. bilateral atDCS vs. sham	Right frontopolar cortex	1 mA, 30 min	Improved semantic word generation during uni- and bilateral atDCS
Ross et al., 2011	*N* = 20 (15 younger and 14 older subjects)	55–69	Randomized cross over within subjects (two age groups)	Picture naming (faces and places, declarative memory retrieval)	atDCS over left or right anterior temporal lobe vs. sham	Contralateral cheek	1.5 mA, 15 min	Improved memory retrieval for faces during left atDCS and for places during right atDCS
Sandrini et al., 2014	*N* = 36 (12 per group)	Mean age ± SD: 67.17 ± 3.68	Randomized between subjects (three stimulation groups)	Episodic memory (memory reconsolidation)	atDCS over left DLPFC + reminder or atDCS over left DLPFC + no reminder vs. sham + reminder	Right frontopolar cortex	1.5 mA, 15 min	Improved memory performance one day and one month after atDCS (with and without reminder)
Park et al., 2014	*N* = 40 (20 per group)	Mean age ± SD in groups with older subjects: 70.1 ± 3.4, 69.4 ± 3.1	Randomized between subjects (two stimulation groups)	(Verbal) working memory	Bilateral atDCS over DLPFC + cognitive training vs. sham + cognitive training	Contralateral arm	2 mA, 30 min, 10 consecutive sessions	Improved working memory one day, seven days, one month after cognitive training combined with atDCS
Zimerman et al., 2013	*N* = 53 (24 younger and 29 older subjects)	55–88	Randomized cross over within subjects	Procedural memory (finger tapping)	atDCS over contralateral (left) motor cortex vs. sham	Right frontopolar cortex	1 mA, 20 min	Improved motor skill learning in older subjects with effects lasting for 24 h

*^∗^Position of “reference electrode” indicates the position of the electrode whose effects are not the focus of the study. In studies, in which atDCS was applied unilaterally, the “reference electrode” is the cathode, whereas it is the anode during ctDCS. To avoid effects of the reference, this electrode is in general enlarged compared to the “stimulation electrode.” The reference electrode can have a cephalic (e.g., contralateral frontopolar cortex) or extracephalic position (e.g., right shoulder). In the study by Boggio et al. (2010), there is no “reference electrode,” because the authors used bilateral tDCS, with equal size, over brain areas of interest in the study.*

The ability to remember the location of an object is important for everyday life. This function (the so-called object-location memory) is known to decline with advancing age and early in the course of dementia. In a recent study (Floel et al., 2012), we focused on object-location memory and investigated whether atDCS (1 mA, 20 min) over the right temporoparietal cortex improves the learning of object-location associations and its retention in healthy older subjects. Subjects were presented with a street map on which buildings were shown at different correct and incorrect positions. In this “statistical” learning paradigm (“LOCATO,” see also Kulzow et al., 2014), the “correct” position of a building occurred more frequently (10 times more often than any of the incorrect positions, which were shown only once) over the course of five training blocks. Outcome measures were learning success at the end of each session (after atDCS and sham) as well as immediate and delayed free recall one week later. We found that subjects in the atDCS condition compared to the sham condition did not differ in learning success and immediate recall but showed memory improvement for delayed recall. That is, one week after atDCS subjects remembered more positions correctly (Floel et al., 2012).

Since impaired semantic word retrieval is also an early marker of age-related cognitive decline (Taler and Phillips, 2008), we also tested the effect of atDCS (1 mA, 20 min) over the left inferior frontal gyrus on the performance in a semantic word generation task (Meinzer et al., 2013). To also investigate the influence of tDCS on task-related brain activity and resting state functional connectivity, subjects were tested in a 3T fMRI scanner (for a detailed description of the setup, see Meinzer et al., 2014b). During sham stimulation, older subjects compared to younger controls showed reduced performance in the semantic word generation task together with increased (compensatory) bilateral prefrontal activity. atDCS significantly improved performance in older subjects up to the level of the younger controls and also led to reduced task-related activity in the bilateral prefrontal cortex, the anterior cingulate gyrus, and the precuneus. In addition to task-related fMRI, we also investigated resting state functional connectivity. The analysis of resting state functional connectivity makes use of the observation that spontaneous fluctuations in the fMRI signal occur synchronously within neural networks (Biswal et al., 1995; Smith et al., 2012). Some of these networks are “task-positive” (i.e., activated by tasks) and reflect sensory and motor systems or networks involved in cognition such as the cognitive control network, while other networks are “task-negative,” which means that they are deactivated during externally driven cognition. The primary task-negative network, the default mode network (DMN) comprises the precuneus/posterior cingulate cortex and the medial prefrontal cortex. Interestingly, some of these brain regions also participate in memory retrieval (Wagner et al., 2005) and it has been found that the DMN is disrupted in patients with AD (Greicius et al., 2004). In line with the literature, we found increased (i.e., compensatory) resting state functional connectivity in task-positive fronto-temporal and medial frontal brain regions during sham. Functional connectivity in posterior regions including the precuneus and the posterior cingulate gyrus (i.e., the DMN) was reduced however. With regard to the atDCS application, we observed that atDCS partially reverses functional connectivity into a more “youth-like” connectivity pattern (Meinzer et al., 2013).

It has to be noted that different studies in older subjects used very different stimulation protocols. Most studies used unilateral atDCS with a “reference” electrode placed over the frontopolar cortex. Some authors also applied bilateral anodal stimulation with an extracephalic reference electrode montage. Using a bilateral stimulation protocol with an extracephalic reference, however, might lead to a significantly different current flow compared to the majority of tDCS studies that used a cephalic reference electrode montage (for a review, see Nitsche and Paulus, 2011). In our group, we compared the impact of unilateral atDCS over the motor cortex to the impact of bilateral atDCS on functional connectivity during a motor task (Lindenberg et al., 2013) and during semantic word generation (Meinzer et al., 2014d). During unilateral stimulation, the anode was placed over the left motor cortex (M1), while the cathode was placed over the contralateral frontopolar cortex. During bilateral motor cortex stimulation, the anode was placed again over left M1, while the cathode was placed over right M1. Bilateral tDCS was intended to upregulate excitability of M1 underneath the anode while concurrently downregulating M1 on the contralateral side. Both unilateral and bilateral tDCS enhanced semantic word generation compared to sham. At the same time, downregulation of activity in frontal regions indicated more efficient processing in both tDCS conditions compared to sham (Meinzer et al., 2014d). Moreover, resting state functional connectivity analysis demonstrated complex interhemispheric modulations of neural networks including the result that bilateral but not unilateral tDCS enhanced connectivity of the posterior cingulate cortex (Lindenberg et al., 2013).

Some studies also investigated the effect of repeated tDCS sessions on cognitive functions (e.g., Meinzer et al., 2014a; Park et al., 2014; Jones et al., 2015b). In the study by Meinzer et al. (2014a), for instance, subjects acquired a novel vocabulary (familiar and novel object picture non-word pair associations) over five consecutive days and received either atDCS over the left posterior temporo-parietal junction or sham tDCS. atDCS yielded steeper learning curves and enhanced learning success at the end of the training (during recall and recognition). In addition, beneficial atDCS effects were maintained until the follow-up assessment one week later. Park et al. (2014) as well as Jones et al. (2015b) also found improved working memory capacities one month after 10 consecutive trainings sessions.

In most studies, tDCS was applied during a specific task. That is, most studies investigating tDCS induced memory formation applied tDCS already during the encoding phase. Nevertheless, most studies still observed an effect on retrieval one week or one month later (e.g., Floel et al., 2012). Sandrini et al. (2014), specifically applied tDCS in the reconsolidation phase in which subjects saw reminders of the previously learned material. Moreover, Fertonani et al. (2014) compared online and oﬄine effects of atDCS on declarative memory retrieval (i.e., they used atDCS during and before the execution of a naming task) in older and younger adults. The authors found beneficial effects of tDCS in older subjects only in the online stimulation condition, whereas in young subjects both stimulation conditions improved retrieval performance. These findings indicate that in aging adults, the cerebral network dedicated to lexical retrieval processing is only facilitated if stimulation is applied to an already “active” neural network (Fertonani et al., 2014).

### tDCS Studies in Patients with AD and MCI

Alzheimer’s dementia (AD) is a progressive neurodegenerative disorder that most often starts with decline in learning and memory formation, but eventually affects attention, executive control functions, reasoning, and language as well (Reitz et al., 2011). Most prominently, the progression of AD has been causally associated with an abnormal accumulation of neurofibrillary tangles and amyloid beta-proteins (Jack et al., 2013). Changes in structural and functional brain architecture in the disorder are accompanied by alterations in cholinergic and glutamatergic neurotransmitter systems (see Francis, 2005).

Since tDCS modulates cortical excitability and induces effects that resemble synaptic plasticity of glutamatergic connections (namely, synaptic LTP and LTD), which can outlast the duration of stimulation for some hours (Bindman et al., 1964; Nitsche et al., 2003), atDCS might be particularly suited for treating AD patients (Floel, 2014; Kuo et al., 2014). It has also been found that atDCS strengthens the connections between remote, but functionally associated brain areas (Polania et al., 2011; Meinzer et al., 2012). Until now, however, only few studies have probed the interventional potential of tDCS for the treatment of AD. As we will describe in greater detail in the following section, these studies have either targeted the temporal cortex because of its important role in memory consolidation or the DLPFC because of its important role in executive control functions, working memory, and memory encoding.

Ferrucci et al. (2008) investigated the effect of atDCS over the temporoparietal cortex on episodic memory and selective attention in 10 patients with probable AD (probable AD = AD diagnosed based on the diagnostic criteria from the National Institute of Neurological and Communicative Disorders and Stroke and the AD and Related Disorders Associations (NINCDS–ADRDA; Mckhann et al., 1984). Here, atDCS was applied over the left temporo-parietal cortex bilaterally (i.e., with the anode placed over the left side and the cathode placed over the right side) and compared to ctDCS and sham stimulation in three different sessions in a randomized cross-over design. The direct current was applied with a strength of 1.5 mA and a duration of 15 min. Performance in a word recognition memory task and a task measuring selective attention was tested before and 30 min after tDCS application. The authors found a significant increase of recognition memory accuracy after atDCS application and a significant decrease after ctDCS was given, whereas sham tDCS led to no change in episodic memory performance. In contrast to these effects on memory performance, the authors did not find changes in visual attention (Ferrucci et al., 2008). In a similar study by Boggio et al. (2009), 10 AD patients received three sessions of tDCS with an intensity of 2 mA for 30 min: atDCS over the left DLPFC, atDCS over the left temporal cortex, and sham stimulation. During each session, patients performed a selective attention task (Stroop), a working memory task (Digit Span), and an episodic memory task (Visual Recognition Memory). Patients showed improved performance in the episodic memory task during atDCS over both the DLPFC as well as the temporal cortex, while no significant changes in attention and working memory (Stroop and Digit Span) could be noted (Boggio et al., 2009).

Ferrucci et al. (2008) and Boggio et al. (2009) demonstrated that temporal atDCS enhances verbal and visual recognition memory, however, sample size in both studies (*n* = 10 patients, no control group) was rather small. Nevertheless, these initial results are very promising. Since effects could already be demonstrated after a single tDCS session, repeated or daily applications over a longer intervention period (e.g., in home-based settings) might induce even greater improvements. This latter issue was addressed in a study by Boggio et al. (2012) examining longer-lasting and thus clinically more meaningful effects of repeated tDCS sessions on episodic memory. In this study, 15 AD patients received five consecutive sessions of atDCS compared to sham stimulation. Stimulation was delivered bilaterally via two electrodes placed over the temporoparietal cortex with a current of 2 mA for 30 min. After atDCS, performance in a visual recognition memory task significantly improved, while even a small decline was noted after sham stimulation. Notably, the atDCS effect persisted for at least four weeks after intervention. Again, no effect with respect to general cognitive performance (here assessed via a visual attention task) was found in line with Ferrucci et al. (2008) and Boggio et al. (2009).

All three studies (Ferrucci et al., 2008; Boggio et al., 2009, 2012) showed specific effects of temporal atDCS on recognition memory, that is, the authors did not find effects on other functions examined such as working memory and selective attention. In the studies by Ferrucci et al. (2008) and Boggio et al. (2012) a stimulation effect was found after stimulation (and even four weeks after the intervention), whereas Boggio et al. (2009) investigated cognitive performance directly during stimulation. Boggio et al. (2009) found enhanced episodic memory performance when stimulating the temporal cortex and also when stimulating the DLPFC. Hence, it is unclear which cognitive processes were actually targeted and whether memory storage *per se*, or consolidation, learning strategies, encoding, or retrieval processes had been enhanced.

As we already discussed for tDCS studies in healthy older subjects, studies in patients with AD used different stimulation protocols as well. While some authors used unilateral atDCS with the reference electrode (i.e., the cathode) over the contralateral frontopolar cortex (e.g., Boggio et al., 2009), others used bilateral stimulation with two electrodes placed over the scalp (anode and cathode) and an extracephalic reference electrode placed over the deltoid muscle, to avoid bias arising from the use of two electrodes with opposite polarities over the scalp (Ferrucci et al., 2008; Boggio et al., 2012). Ferrucci et al. (2008) applied 1.5 mA for 15 min, whereas Boggio et al. (2009, 2012) stimulated with 2 mA for 30 min.

Khedr et al. (2014) further investigated the potential of repeated prefrontal tDCS sessions to beneficially modulate functional scores for patients with AD. In this randomized controlled trial (RCT), 34 patients were randomly assigned to three groups in which they either received atDCS or ctDCS (with the anode or cathode placed over the left DLPFC) or sham tDCS. Stimulation was applied for 25 min at 2 mA on 10 consecutive days. The impact of prefrontal tDCS was investigated with regard to clinical scores and general cognitive abilities using the mini-mental state examination (MMSE; Folstein et al., 1975) and the Wechsler adult intelligence scale (WAIS-III; Wechsler, 1997). The authors demonstrated that clinical scores measured with MMSE and WAIS-III improved significantly directly after prefrontal atDCS or ctDCS compared to sham stimulation. Effects on some subscales (e.g., Digit Span, Vocabulary) even lasted for two months after treatment. With regard to the neurophysiologic mechanisms, the authors also measured a significant reduction in the latency of auditory P300 evoked potentials which is known to be pathologically increased in AD and has been suggested as a biological marker (Parra et al., 2012). Additional assessments on cortical excitability operationalised via the “Cortical Silent Period,” which indicates GABA_B_-receptor activity (Paulus et al., 2008), before and after all tDCS sessions, did not reveal any significant alterations though.

In another RCT, Cotelli et al. (2014) combined prefrontal tDCS with a computerized face-name association memory training. In this study, 36 AD patients were randomly assigned to three groups and received either atDCS over the left DLPFC or sham tDCS in combination with the memory training or received atDCS over the left DLPFC in combination with a motor training. tDCS was applied with 2 mA for 25 min. After two weeks of daily training (10 trainings sessions in total), the authors found an effect of memory improvement which diminished after three and six months. The combination of memory training with atDCS, however, was not superior to sham stimulation combined with memory training.

Moreover, Suemoto et al. (2014) investigated the effects of repetitive sessions of atDCS over the left DLPFC on apathy measured with the Apathy Scale (Starkstein et al., 1992) answered by a caregiver of the patient. Apathy is considered as the most common neuropsychiatric symptom in AD caused by disease-related changes of neural activity in prefrontal neural circuits. Secondary outcomes included depression, caregiver burden, clinical scores measured by the cognitive subscale of the Alzheimer’s Disease Assessment Scale (ADAS-Cog; Mohs et al., 1997) including tasks testing for memory performance, language, praxis, and orientation, which are referred to as the core symptoms of AD. Forty patients were randomly assigned to two groups either receiving six sessions with 20 min of 2 mA atDCS or sham stimulation for two weeks. The authors, however, neither found differences between the groups in apathy nor in any of the secondary outcome measures.

Mild cognitive impairment is a brain functional syndrome describing the onset and evolution of cognitive impairments beyond those expected when considering age and education of the individual. Cognitive impairments can be objectively measured but are still not significant enough to interfere with daily activities (Petersen and Negash, 2008). Although MCI can be present with a variety of symptoms, in most cases memory loss is the predominant symptom in MCI (amnestic and amnestic plus MCI, see Petersen and Negash, 2008). Since it has been shown that individuals with MCI progress to AD at a rate of approximately 10–15% per year (Grundman et al., 2004), MCI can be seen as a prodromal stage of AD.

Given the fact that brain damage in AD may be too severe to be treated successfully, research now increasingly focuses on patients with MCI as a transitional stage between healthy and pathological aging. Most important this stage of “pre-dementia” might also be more amenable to disease-modifying interventions (Langbaum et al., 2013). Thus, MCI constitutes an area of highest relevance for the application of non-invasive brain stimulation alone and in combination with other interventional approaches such as cognitive training (for reviews about other non-pharmacological interventions in patients with memory decline, see also Cotelli et al., 2012; Elder and Taylor, 2014). The impact of tDCS on cognition in MCI patients, however, has not been explored so far.

Based on our work on healthy older adults mentioned above (Meinzer et al., 2013), we applied atDCS over the left inferior frontal cortex during semantic word generation in 18 patients with MCI compared to healthy controls. Similarly to our study in older healthy participants, atDCS significantly improved semantic word generation to the level of healthy controls compared to sham. In this study, we also used fMRI to investigate the effect of atDCS on task-related brain activity and resting-state functional connectivity. We demonstrated that atDCS reduces task-related prefrontal hyperactivity and resulted in a normalization of abnormal network configuration during resting-state fMRI (Meinzer et al., 2014c).

At clinicaltrials.gov, the largest database of clinical trials run by the United States National Library of Medicine at the Institute of Health, only a few more ongoing tDCS studies in patients with AD and MCI are currently listed. These studies, for instance, focus on repeated and daily stimulation over a longer period of time in home-based settings and investigate changes in multiple clinical outcome measures including quality of life and physical activity. While information about ongoing studies retrieved from the clinical trials database reflects current trends in the field, information might not be complete and should be treated with caution though.

## Methodological Considerations and a Discussion of Study Protocols

An important issue that needs to be taken into consideration when comparing tDCS studies is the electrode montage and the use of terms such as “cathodal” and “anodal” stimulation. As mentioned already in the introductory part of this review, an anode and a cathode are always required during tDCS application to deliver the current to the brain. Thus, it is not possible to “only” apply anodal or cathodal stimulation. In addition to the intended effect of atDCS on one specific brain region, there might always be an inhibitory effect of the cathode. Therefore, it is important to emphasize that the “site of stimulation” is not simply the location of one electrode but rather the combination of anode and cathode. As a consequence, it can be difficult to ascribe any observed behavioral effects to a specific stimulation site as the current passes through the brain and also modulates neural activity (to a lesser extent) in other brain regions.

On the other hand, even though previous modeling studies have shown that atDCS results in modulation of large cortical areas (e.g., Wagner et al., 2007), this does not necessarily result in an unspecific upregulation of task-related activity patterns. Rather, studies combining behavioral outcome measures with task-related fMRI suggest that during task performance enhanced connectivity in a given network provides the basis for enhanced neural efficiency in highly specific brain areas critical for task performance (Holland et al., 2011; Meinzer et al., 2012).

In the present review, a distinction was made between stimulation paradigms that place one electrode (in almost all cases the anode) over a specific target area (i.e., the temporoparietal cortex or the DLPFC) and the cathode over a “reference” region (usually the contralateral frontopolar cortex), studies that place both anode and cathode over the target area bilaterally, and paradigms that use an extracephalic reference electrode montage (e.g., Ferrucci et al., 2008; Boggio et al., 2012; cf. **Tables 1** and **2**). The latter protocol might avoid some of the shortcomings caused by a cephalic reference montage such as unwanted reversed effects of tDCS under the reference electrode which might be of special importance in clinical settings when a homogeneous shift of cortical excitability is needed (Nitsche et al., 2007). However, it is unclear whether studies are comparable (for a review, see Nitsche and Paulus, 2011). It should be noted that one study by Vernieri et al. (2010) found decreases in cerebral vasomotor reactivity (cVMR), that is, in the autonomic ability of cerebral arterioles to dilate following a vasodilatory stimulus, with an extracephalic reference electrode. The same extracephalic reference electrode, for instance, was used in the studies by Ferrucci et al. (2008) and Boggio et al. (2012) in AD patients. An equal decrease in cVMR was not observed in a carefully controlled study by List et al. (2015). Hence, for safety concerns, it is strongly recommended to use the cephalic reference electrode montage in any population with potential vulnerability to changes in cerebral autoregulation.

**Table 2:** Overview of tDCS studies in AD and MCI patients.

Reference	Number of subjects	Trial type	Modalities	Polarity of stimulation electrode, position	Position of reference electrode	Current strength, duration, number of consecutive sessions	Outcome
Boggio et al., 2009	*N* = 10	Randomized cross over within subjects	Episodic memory (memory encoding and recognition), working memory, selective attention	atDCS over left temporal cortex or left DLPFC vs. sham	Right frontopolar cortex	2 mA, 30 min	Improved recognition during temporal and prefrontal atDCS
Boggio et al., 2012	*N* = 15	Randomized cross over within subjects	Episodic memory (memory encoding and recognition), selective attention	Bilateral atDCS over temporoparietal cortex vs. sham	Deltoid muscle	2 mA, 30 min, five consecutive sessions	Improved recognition at the end of treatment day five, one week later, and one month later
Cotelli et al., 2014	*N* = 36 (12 per group)	Randomized between subjects (three stimulation groups)	Episodic memory (encoding and retrieval)	atDCS over left DLPFC + memory training or atDCS over left DLPFC + motor training vs. sham + memory training	Deltoid muscle	2 mA, 25 min, 10 consecutive sessions	No additive effects of atDCS on memory performance when combined with memory training
Ferrucci et al., 2008	*N* = 10	Randomized cross over within subjects	Episodic memory (encoding and recognition), cued choice response time task	Bilateral atDCS or ctDCS over temporoparietal cortex vs. sham	Deltoid muscle	1.5 mA, 15 min	Improved recognition 30 min after atDCS
Khedr et al., 2014	*N* = 34 (11/12 per group)	Randomized between subjects (three stimulation groups)	General cognitive abilities/clinical scores, motor cortex excitability, event related potentials (P300)	atDCS or ctDCS over left DLPFC vs. sham	Right frontopolar cortex	2 mA, 25 min, 10 consecutive sessions	Improved clinical scores and shorter P300 latency after atDCS and ctDCS, one and two months later
Meinzer et al., 2014c	*N* = 18 MCI patients	Randomized cross over within subjects	Semantic word generation	atDCS over left inferior frontal gyrus vs. sham	Right frontopolar cortex	1 mA, 20 min	Improved semantic word generation (less errors) during atDCS and normalized functional network configuration
Suemoto et al., 2014	*N* = 40 (20 per group)	Randomized between subjects (two stimulation groups)	Apathy, general cognitive abilities, depression	atDCS over left DLPFC vs. sham	Right frontopolar cortex	2 mA, 20 min, six consecutive sessions	No atDCS effects

*The only study that tested MCI patients is highlighted by a gray field.*

Another issue is the fact that the most effective stimulation parameters for enhancing cognitive function in older subjects and patients with AD and MCI are still unclear. In the studies presented here, the current strength varied from 1 to 2 mA and the durations ranged from 6 to 30 min. Although stimulation with a stronger current over a longer period of time is more intense, it is unknown whether stimulation is also more effective. Investigating the problem of whether a greater effect can be achieved by stimulating with stronger currents, Nitsche and Paulus (2000) showed that a facilitation of excitability induced by atDCS applied with 1 mA over the motor cortex can be prolonged using currents of 2 mA. A recent study by Batsikadze et al. (2013), however, demonstrated that ctDCS over the motor cortex with a current strength of 2 mA reverses the “usual” 1 mA ctDCS specific inhibitory effect to facilitation. Results suggest that a higher tDCS intensity does not necessarily increase efficacy of stimulation but might shift the direction of excitability alterations. Furthermore, most effects are also described for stimulation periods of up to 20 min. Thus, it is also unclear whether longer-lasting stimulation has greater effects or is detrimental.

As already mentioned, some authors found a stimulation effect when patients performed a task during or shortly after stimulation (“online-effects;” e.g., Boggio et al., 2009), while others report improvements several days or even weeks later (“oﬄine-effects,” e.g., Ferrucci et al., 2008; Boggio et al., 2012). Investigating the question of timing of tDCS, Stagg et al. (2011) conducted a series of behavioral motor learning experiments in young healthy controls. Application of tDCS during an explicit sequence-learning task led to modulation of behavior in a polarity specific manner: relative to sham stimulation, atDCS was associated with faster learning and ctDCS with slower learning. Application of tDCS prior to performance, however, led to slower learning after both atDCS and ctDCS. The authors interpret these results as being consistent with the notion that atDCS induced LTP-like changes in interneurons of the primary motor cortex having the potential to destabilize the cortical network. In order to prevent destabilization and to maintain neural activity in a useful range, regulatory metaplasticity mechanisms have been proposed that might lead to a time-dependent interaction of atDCS and motor learning (Stagg et al., 2011). In a recent meta-analysis, Hsu et al. (2015) compared effects of tDCS in older subjects and patients with AD with regard to the question whether stimulation was applied online while participants were engaged in a specific task or before task performance. In older healthy subjects, the authors found more prominent effects of atDCS when stimulation was delivered before the execution of the task. In patients with AD, however, more pronounced effects were found when subjects received stimulation during the execution of a task (Hsu et al., 2015). The latter tDCS “online-effects” might be related to membrane depolarization, whereas “oﬄine-effects” additionally involve changes at NMDA receptors and LTP-like mechanisms (Stagg and Nitsche, 2011). With regard to these mechanisms, Hsu et al. (2015) speculate that during healthy aging, subjects may benefit more from LTP-like mechanisms introduced in repeated and multiple sessions. Patients with manifest AD, however, show various structural, metabolic, and functional alterations such as a disruption of synaptic plasticity and an inhibition of LTP (for a review, see Jagust, 2013). Therefore, subjects with AD might rather benefit from online stimulation. Whether this also applies for patients with MCI has not been investigated so far. It has also to be taken into consideration that patients with AD and MCI might display altered neurotransmitter concentrations (Nardone et al., 2011). Cortical excitability in these patients as well as potentials for its modulation should be explored in future studies.

The authors of studies demonstrating effects after a single tDCS session often speculated that repeated or daily applications over a longer intervention period (e.g., in home-based settings) might induce greater improvements. As has been shown in healthy young subjects, repeated stimulation after a short interval of 20 min (i.e., during the after-effects of stimulation) results in initially reduced yet ongoing excitability enhancement (LTP-like plasticity) while temporally contiguous stimulation and repeated stimulation after a prolonged time interval (i.e., after the after-effects have disappeared) might also result in a reversal of neuroplasticity (Monte-Silva et al., 2013). Also, it seems likely that short stimulation intervals during repeated stimulation could lead to saturation or counter-regulatory effects which may prevent the neurons from over-excitation. The study by Monte-Silva et al. (2013), specifically demonstrated that neuronal LTP-like plasticity can be induced by repeated stimulation, and that a specific time window is crucial for its induction. Future experiments could explore the specific mechanisms of action as well as the optimal timing of plasticity induction suited to improve learning and memory performance. Again, these mechanisms have not been investigated in patients with AD and MCI at this point.

In addition to repeated stimulation protocols, pharmacological interventions have been shown to prolong the after-effects of tDCS for up to 24 h after the end of stimulation. As already mentioned, Nitsche et al. (2004) demonstrated in healthy young subjects that D-Cycloserine, a partial NMDA agonist, can be used to consolidate neuronal excitability enhancements induced by atDCS. In addition, it has also been found that dopamine (or the dopamine precursor L-dopa) significantly prolongs the after effects of tDCS (Kuo et al., 2008), although the direction of effects seem to be dose-dependent and far more complex (Monte-Silva et al., 2010). Whether these effects can be replicated in older subjects and patients already experiencing cognitive decline needs to be determined in the future.

As one can see from the review of reports in older subjects and patients with AD and MCI, there is a variety of studies targeting different brain regions and functions. Functions of interest are motor learning, working memory, memory formation (comprising encoding, retrieval, and recognition of episodic memories), declarative memory retrieval, decision making, and error awareness (see **Tables 1** and **2**). Hence, there has not been a systematic investigation into the effects of tDCS on specific cognitive functions and brain regions yet. For instance, in the studies reported, the temporal cortex, the temporoparietal cortex, the DLPFC, inferior frontal cortex, or motor cortex was targeted. This suggests that some of the areas seem to be more inclined to tDCS enhancement than others. As stated in a review by Tremblay et al. (2014), prefrontal tDCS, in particular, has the potential to modulate a broad range of cognitive functions at the same time. A given stimulation protocol, thus, may simultaneously modulate various cognitive functions in similar or opposite directions (i.e., in terms of facilitation or inhibition). Therefore, it is difficult to establish viable predictions.

## Future Directions

The present review of tDCS studies in healthy and pathological aging has shown that there are encouraging results for application of tDCS in patients with AD and MCI. However, there are also many questions that remain to be answered. First of all, the discussion of different protocols of the reviewed studies revealed that stimulation parameters including current strength, duration, and whether stimulation should be applied in a single session or repeatedly within specific time intervals is still unclear. It should also be investigated further which brain regions should be targeted by tDCS stimulation. Another aspect that also needs to be investigated in RCTs is the sustainability of effects comprising measures such as changes in quality of life and the question whether effects can be transferred into daily life. Many of the underlying mechanisms involved in tDCS are still unclear, particularly with regard to the specific populations of older subjects and patients with AD. Surprisingly little is known on the potentials of tDCS in patients with MCI, although in this stage of “pre-dementia” disease-modifying interventions might be most suitable. Up to now, only one study has been published that investigated the effects of tDCS in patients with MCI (Meinzer et al., 2014c). The results of this study, however, are encouraging, since they demonstrate a tDCS-induced improvement of behavioral performance in patients up to the level of healthy controls. Moreover, improved performance was accompanied by a normalization of neural activation during task execution as well as in functional connectivity at rest.

A second issue that should be investigated in the future is the question whether tDCS effects might be further modulated pharmacologically with dopaminergic and serotonergic agents. With regard to the specific populations (older subjects, patients with AD and MCI; all of which often present with antidepressant medication), the effects of serotonergic agents are of particular interest. A study published by Nitsche et al. (2009) showed that motor cortex excitability using tDCS was enhanced by the selective serotonin reuptake inhibitor (SSRI) citalopram (single dose, 20 mg). However, the extent to which SSRIs also impact the effect of tDCS on cognitive tasks remains unclear. In a related study, we are currently investigating the effect of SSRI on atDCS over the temporoparietal cortex during the object location learning task LOCATO (Floel et al., 2012; Kulzow et al., 2014). If succeeding, this combination could be extended to long-term training and tDCS protocols in subsequent studies. Other medications frequently used in AD patients are acetylcholesterinesterase inhibitors. To our knowledge, however, no studies to date have been investigating whether there is an interaction with tDCS application.

Third, it has to be noted that cognitive decline during healthy and pathological aging does not affect all individuals equally. Clear associations exist between the rate and severity of cognitive decline and a variety of individual factors. These factors include, among others, the level of oxidative stress and free radical damage, chronic inflammation processes, imbalanced and declining hormone levels, as well as other medical conditions. The influence of overweight and diabetes, suboptimal nutrition, physical activity, social support, and lifelong cognitive stimulation on aging and cognitive decline has also been reported (for a review, see Mattson, 2015). Several observations suggest that the effect of tDCS protocols may also depend on these individual factors mentioned. Berryhill and Jones (2012), for instance, investigated the impact of atDCS over the DLPFC on working memory in older subjects (see **Table 1**) and found that tDCS was only beneficial in older adults with higher education. In the less educated group, tDCS provided no benefit to verbal or visual working memory performance. The authors interpret these findings as evidence for differential frontal recruitment in both groups as a function of strategy. In a related study, the authors investigated the influence of strategy use and motivation on working memory enhancement. This study also revealed that the use of specific working memory strategies in combination with prefrontal tDCS enhanced working memory performance in individuals with higher working memory capacity. Financial motivation further amplified the working memory enhancing effect of tDCS in both low and high capacity groups (Jones et al., 2015a). These studies provide evidence that individual differences including general information processing capacity, education, motivation, and affect might play an important role which should be considered especially in patients with MCI and AD. Finally, research is needed to investigate the value of additional biomarkers, such as learning relevant candidate genes, inflammatory markers, neurotransmitter concentrations, markers of cortical excitability, and neurodegeneration, as well as neuronal activation patterns (i.e., neural activation during task execution and resting state functional connectivity) in predicting the therapeutic success of tDCS. Taking these individual factors and their interaction with the mechanisms involved in tDCS into account will eventually lead to individually optimized interventional approaches in the future.

With the present review article, we aimed to summarize the existing and ongoing work investigating effects of tDCS in healthy and pathological aging. As we demonstrated in this review, little work has been done so far in MCI patients and only one tDCS study with MCI patients has been published (Meinzer et al., 2014c). This is surprising, since MCI constitutes a prodromal stage of AD in which patients might be most susceptible to plasticity enhancing interventional approaches. From the results of the studies reviewed, we conclude that tDCS is a promising tool that can be used to influence brain activation and thus ameliorate cognitive dysfunction associated with healthy and pathological aging. Specifically, we recommend to use stimulation protocols in which tDCS is applied repeatedly in multiple sessions and within specific time windows (e.g., within the after-effects of stimulation). Patients with AD (who already acquired severe impairments in the course of the disease) seem to benefit more from stimulation during a task, whereas in less impaired subjects neuronal plasticity can also be enhanced when tDCS is applied before task execution. Moreover, the combination of tDCS with pharmacological interventions seems to be an approach well worth pursuing. Since many issues remain to be resolved, we emphasize the need to conduct RCTs to establish the efficacy of individualized stimulation protocols for cognitive scores as well as for outcome measures relevant for daily life activities. Future studies also need to quantify the mechanisms of tDCS-induced neuroplasticity. This is an exciting opportunity to delve into potentials of what is commonly seen as neurodegenerative processes.

## Conflict of Interest Statement

The authors declare that the research was conducted in the absence of any commercial or financial relationships that could be construed as a potential conflict of interest.
